# In and out among the Hospitals

**Published:** 1888-06-30

**Authors:** 


### IN AND OUT AMONG THE HOSPITALS.

BY A SECRETARY OF TEN YEARS' STANDING.

Copies of Annual Reports and Rules, Accounts of Meetings, Changes in the Staff, &c., should be addressed THE EDITOR, The Lodge, Porchester Square, W.

### KING'S COLLEGE HOSPITAL.

THE want of uniformity in the method of keeping hospital accounts is never more apparent than when perusing the report of this hospital. In some measure the system adopted is in accordance with modern ideas, but this, if anything, makes the difficulty of understanding the accounts greater, since the modern system is not carried out in its entirety. In the report before us there are no less than eleven separate accounts to wade through before any idea can be obtained of the financial condition of the hospital. After careful perusal, the conclusion arrived at is that the receipts amounted to £18,721 7*s.* 1*d.* (including legacies and a special donation of £1,000 stock, which do not appear to have been regarded as receipts), and the expenditure £25,477 0*s.* 1*d.*—in this latter item there is a sum of £8,942 4*s.* 10*d.* for extraordinary expenses; so, had it not been considered necessary to incur this exceptional expenditure the balance for the year would have been considerably on the right side, and on this we have to congratulate the charity. The extra expenditure was incurred for new nurses' cubicles rendered necessary since the transfer of the management of the nursing from the St. John's Home to the committee, additional lavatory accommodation to wards, new furniture, linen, etc. As no definite balance-sheet and statement of reserve fund is published among the multifarious other tables, it is difficult to state what were the liabilities and what the assets at the end of the last financial year. The daily average number of beds occupied was 151, and after deducting 1*s.* for each out-patient, the average cost per bed occupied on the ordinary expenditure amounts to £102 15*s.* 11*d.* This is probably the highest rate of expenditure of any general hospital in England. There were 1,811 in-patients, and 17,248 out-patients, casualties and maternity cases, treated during the year. Two donations of £1,000 were received—one from Mr. Matthew Whiting, an old friend of the hospital, and the other was received anonymously. High encomiums of praise are bestowed on Miss Monk, the sister matron, for the assistance she has rendered in reorganising the nursing staff and formulating the scheme for supplying private trained nurses, accommodation for 25 of whom has been supplied in the new nurses' dormitories; and the committee go so far as to say that the plans for the construction of the cubicles and the sanitary improvements in the lavatories have been due in a great measure to Miss Monk's initiative, and further, "that her suggestions for the sanitary arrangements have placed the hospital in the van of such improvements, and that the institution will never cease to owe a great debt of gratitude to her skill and generous labour." The nurses take a prominent part at King's, for the committee record their sense of the "devoted interest" of Sister Sibbald, by whose desire Mr. Whiting's gifts are bestowed on the hospital; and Ward Sister Miss K. Philippa Hicks is welcomed back from Egypt, where she has been doing gallant service with the troops. The nursing staff has been increased, with the result that £262 has been added to the total of salaries for sisters and nurses. A bazaar in aid of the funds was given in the hall of King's College. Her Royal Highness Princess Louise was present, and a sum of £1,352 6*s.* 6*d.* was raised. The following gentlemen have joined the committee: Dr. Stokoe, Mr. G. Wellesley, Mr. A. Brandreth, and Lieut.-Colonel Saner. Mr. W. Watson Cheyne, having filled the post of assistant-surgeon for seven years, has now become a full surgeon, and Mr. Ashley Gibbings has been appointed dental surgeon, in succession to Mr. S. Hamilton Cartwright, who has resigned his appointment, after twelve years' service. The Convalescent Home at Hemel Hempstead is of great service to the hospital, and though the larger number of patients have first been treated at King's, many are admitted from other metropolitan hospitals.

### CITY OF LONDON HOSPITAL FOR DISEASES OF THE CHEST.

The receipts for the year fell short of the payments by £720 16s., the income being only £8,089 7*s.* 3*d.*, and the committee were obliged to dispose of £1,506 worth of stock to meet current expenses and to pay off a loan on the bankers. No balance-sheet is published in the report, nor is there any statement of the liabilities of the charity, but it is certain that further support is much needed. After deducting 1*s.* per head for each out-patient, the cost per bed occupied amounts to £84 5*s.*, which cannot be considered a high figure when the nature of the case treated is taken into account, so much nourishment being needed for most patients suffering from chest diseases. Annual subscriptions show a steady yearly increase, and this may be taken as an index of the confidence of the public in the hospital. Dr. Angel Money resigned his appointment as one of the assistant physicians, and the committee appointed Dr. T. Glover Lyon to fill the vacancy that was thus caused. The Kyrle Society has been active in organising concerts for the amusement of the patients, and other friends have also assisted in a similar way. No charity needs more the assistance of a Samaritan Fund than this does, and it is much to be regretted that the receipts for the fund are so small, the total at the disposal of the committee last year being only £41 19*s.* 4*d.*

### THE SUFFOLK GENERAL HOSPITAL.

At this county hospital the number of patients treated in the wards last year was 391, and there were besides 1,092 out-patients. The number of in-patients is below the average, owing to two of the wards having been closed for repairs. The daily average number of beds occupied was only 47. The total receipts for the year amounted to £2,780 12s., which is an increase of £54 on the preceding twelve months, although there was a falling off in subscriptions and church collections. There was a total expenditure of £2,768 17*s.* 2*d.*, of which a large sum was expended in repairs; but it is curious to note that there was an increase of £14 15*s.* 2*d.* in the household expenses, notwithstanding, that the number of patients under treatment was considerably less. After deducting 1*s.* for each out-patient, the cost per bed occupied is £57 19*s.* 3*d.* To commemorate the Queen's Jubilee the committee appealed for funds for facing the walls of the two lower wards with tiles, and to form a retiring fund for nurses. The appeal produced the sum of £487 2*s.* 6*d.* out of which £482 0*s.* 5*d.* was expended on the tiles, and the balance, £5 2*s.* 1*d.* was credited to the nurses' proposed pension fund. The last-mentioned sum will not go far towards providing pensions, &c. It may have been considered, now that the nurses can join the "National Pension Fund for Nurses," the very smallest portion possible was enough to satisfy those who are in favour of establishing a special pension fund in the Suffolk Hospital. At the last general meeting three names were added to the list of vice-presidents, by the election of R. Macnab, Esq., M.D., W. E. Image, Esq., and J. G. Barclay, Esq. The late matron, Mrs. Robinson, who was elected in 1884, resigned on the 17th May last. Under her management, many changes and improvements were introduced, tending to increase the comfort of the patients and nurses. Miss Shaw was elected to fill the vacancy thus created. It is a pity that the report is published in such an unwieldy form, it is on large-sized foolscap and partakes of none of the neatness that characterises the reports issued by the leading provincial hospitals.

